# Associations between Prenatal Education, Breastfeeding and Autistic-Like Behaviors in Pre-Schoolers

**DOI:** 10.3390/children8020124

**Published:** 2021-02-09

**Authors:** Jing Chen, Esben Strodl, Li-Hua Huang, Jing-Yi Chen, Xin-Chen Liu, Jian-Hui Yang, Wei-Qing Chen

**Affiliations:** 1 Department of Epidemiology, School of Public Health, Sun Yat-Sen University, Guangzhou 510080, China; chenj278@mail2.sysu.edu.cn (J.C.); hlihua2@mail2.sysu.edu.cn (L.-H.H.); chenjy246@mail2.sysu.edu.cn (J.-Y.C.); liuxch57@mail2.sysu.edu.cn (X.-C.L.); yangjh83@mail2.sysu.edu.cn (J.-H.Y.); 2 School of Psychology and Counselling, Queensland University of Technology, Brisbane 4059, QLD, Australia; e.strodl@qut.edu.au; 3 Department of Information Management, Xinhua College of Sun Yat-Sen University, Guangzhou 510080, China

**Keywords:** prenatal education, autistic-like behaviors, breastfeeding, preschool children

## Abstract

This study aimed to investigate the association between prenatal education and autistic-like behaviors of preschoolers as well as the mediating role of breastfeeding in their associations. A cross-sectional study via a structured questionnaire was conducted with 67,578 preschool children and parents from Longhua District of Shenzhen, China. Hierarchical logistic regression models were performed to explore the associations between maternal participation in prenatal education and autistic-like behaviors in preschoolers, as well as the mediating effect of breastfeeding in the associations. After controlling for potential confounders, logistic regression analysis indicated that maternal participation in prenatal education could significantly increase the prevalence of breastfeeding, and the strength of the association was enhanced with the increase frequency of prenatal education visits (ORs ranging from 1.191 to 1.899). While both maternal participation in prenatal education (ORs ranging from 0.732 to 0.798) and breastfeeding (OR = 0.773) were significantly associated with the lower presence of autistic-like behaviors in preschoolers. The crossover analysis indicated that children with both maternal prenatal education and breastfeeding had the lower risk of presence of autistics-like behaviors (OR = 0.569). Furthermore, mediation analysis illustrated that breastfeeding mediated the association between maternal participation in prenatal education and the presence of autistic-like behaviors in preschoolers, with a mediating effect of approximately 14.3%. Our findings suggest that maternal participation in prenatal education is significantly associated with a decreased risk of autistic-like behaviors in preschool children through increased breastfeeding in the mothers who attended prenatal education.

## 1. Introduction

Autism spectrum disorder (ASD) is a pervasive developmental disorder characterized by social impairment, restrictive interests and cognitive inflexibility, with its main symptoms including social communication barriers, language barriers and repetitive stereotyped behavior [1]. Usually, ASD is manifested before the age of three, but most children with ASD are not clinically diagnosed until the age of three or later [2]. Autistic-like behaviors, an important clinical manifestation for the diagnosis of ASD, include social interaction and communication disorders [3,4], language development disorders [5], repetitive behaviors and parochial interests [6,7]. Currently, one in every 59 children in the United States is diagnosed with ASD [8], and approximately 52 million people worldwide suffer from ASD [9]. Due to the lack of a national monitoring system, there has been no large-scale epidemiological survey to address the prevalence of ASD in China to date [10]. But several regional studies reported relatively low prevalence rates of ASD. For instance, a systematic review revealed that the prevalence of ASD was 2.64% in China [11]. Another recent meta-analysis showed a pooled prevalence of ASD in China of 39.23 per 10,000 [12]. However, Constantino’s and Charman’s findings also indicate that autistic traits are continuously distributed in the general population [13].

The prior three studies found an association between earlier autistic traits and later emotional or behavioral problems in different developmental stages, i.e., from preschoolers at age 14 to 15 months to 3 years old [14], from age 2 to age 4 [15] and from preschoolers at age 5 to school-age children at age 7 as well [16]. As such, without timely intervention, autistic-like behaviors in preschoolers are a significant risk factor for the development of serious cognitive and learning deficits and lifelong disability with poor prognosis [17]. Given the high prevalence of ASD and autistic like behaviors in children, and the trajectory of impaired development, there is a need to further identify modifiable risk factors for autistic like behaviors in young children. Identifying such modifiable risk factors will help guide public health interventions aimed at reducing the prevalence of ASD and autistic like behaviors in children. 

Prenatal education is a vital component of maternity care provided by public health departments, hospitals, midwives and private and community agencies for mothers and their partners [18]. Traditionally, prenatal education has occurred as a one-on-one interaction between a clinician and individual woman or a formal childbirth education class, but a group model of prenatal care, Pregnant Women School, has been widely implemented since 1995 [19,20]. Prenatal education imparts knowledge about pregnancy, childbirth, puerperium and neonatal care to pregnant women [21]. In each class, the clinicians may give a lecture on a special topic for pregnant women, such as proper maternal and infant nutritional diets or maternal weight management during pregnancy, guide pregnant women in how to breastfeed and its benefits, and so on [22]. Prenatal education can not only improve maternal self-care, management and awareness about pregnancy, but also promotes short term maternal and child health [20,23]. Unfortunately, research on the long-term effects of prenatal education is lacking. This is especially the case for improvements in the long-term physical and mental health of children caused by mothers’ changing their behaviors due to prenatal education.

There is good evidence that prenatal education can increase the rate of breastfeeding by providing appropriate knowledge and skills [24,25,26]. In accordance with these findings, Schmidt et al. found that prenatal education could increase breastfeeding initiation among low-income women, especially non-Hispanic white and black women [27]. These findings are important as there is also emerging evidence that breastfeeding is a protective factor for childhood cognitive development. For example, Horta et al. reinforced the evidence that breastfeeding was positively associated with an intelligence quotient in childhood [28]. Moreover, three recent studies reported that exclusive breastfeeding was associated with lower risk of ASD [29,30,31], and a systematic review and meta-analysis indicated that breastfeeding could significantly protect against ASD [32].

Whether prenatal education can reduce the risk of autistic-like behaviors of children, whether this could be due to increased breastfeeding and whether prenatal education and breastfeeding have combination effect on autistic-like behaviors, remains unclear. Therefore, this study aimed to resolve these issues. 

## 2. Materials and Methods

### 2.1. Sample and Recruitment

Participants of this study were recruited from the Longhua Child Cohort Study (LCCS), which aimed to estimate the effect of early-life family and school risk and protective factors upon children’s psycho-behavioral development [33,34,35,36]. In October 2017, a total of 67,861 child-caregiver dyads were approached, and 67,578 child-caregiver dyads were included in our analysis after excluding those with incomplete records. This study was approved by the Institutional Review Board of the School of Public Health at Sun Yat-sen University (ethics clearance No.: 2015–2016), and all adult participants provided their informed consent.

### 2.2. Data Collection

A structured questionnaire was used to collect the parents’ socio-demographic characteristics (including age, education level, marital status and family economic income), the child’s demographic information (including date of birth, gender, single child or not, etc.), parental participation in prenatal education, the mother’s breastfeeding history and the child’s current autistic-like behaviors. The investigators trained the health care doctors and kindergarten teachers of the participating child-care institutions in how to give the questionnaire to parents and guided them in filling in relevant information and questionnaires. The questionnaire had to be filled with the child’s parent or child caregiver of more than 1 year, and the child’s mother was preferred. The investigators checked the completed questionnaire and asked the parents to complete any missing items. After the data was entered into a database then 10% of the responses were randomly selected and compared against the answers in the hardcopy questionnaires, and any input errors were corrected. 

### 2.3. Measurement of Prenatal Education, Breastfeeding and Autistic-Like Behaviors

Maternal participation in prenatal classes was measured with the following two questions. “Whether did you attend the Pregnant Women School?” Response options were “Yes” or “No”. If the participants answered “Yes”, they were further asked “How many times did you attend Pregnant Women School?” Response options were categorized into once (scored 1), twice (scored 2), three times (scored 3), four times and over (scored 4). In the final data analysis, parents attending the Pregnant Women School were categorized into five groups (never, once, twice, three times and ≥ four times). Mothers who reported having breastfed were asked to report whether this involved exclusive breastfeeding (i.e., feeding their child only with breastmilk and not formula or other foods/drink) or mixed feeding (i.e., feeding their child with both breastmilk, formula and other foods/drink). Autistic-like behaviors were measured by the Autism Behavior Checklist (ABC), a scale for ASD screening and diagnosis comprised of five subscales (sensory ability, communicative ability, motor ability, language ability and self-care ability) with a total of 57 items [37]. Each item is weighted with score from 1 to 4, and the total score was 158 points. We used established scoring criteria for China, with a total score of ≥ 31 points indicating the presence of autistic-like behaviors; and a total score ≥ 62 points indicating the presence of a diagnosis of ASD [38]. In our study, a cut-off of ≥ 31 was chosen to distinguish preschool children with autistic-like behaviors.

### 2.4. Data Analysis

Regarding data analysis, means and standard deviations (X ± SD) were used to describe continuous variables, and proportions were used to describe categorical variables. Chi-square tests, Student’s t-tests and ANOVA were employed to test the associations between the different socio-demographic characteristics, prenatal education attendance and the prevalence of autistic-like behaviors in preschoolers. Binary logistic regressions were performed to investigate the associations between the dependent variable of the presence of autistic-like behaviors in preschoolers with the independent variables (attendance at prenatal education, and breastfeeding patterns) after adjusting for potential confounders of child’s age, gender, parents’ education level, family income and parental marital status.

Moreover, we made a crossover analysis to assess combination effects of maternal prenatal education and breastfeeding on the presence of autistics-like of behaviors in pre-schoolers, and the parents attending the Pregnant Women School were then categorized into two groups (never vs. once and over). The multiplicative interaction was estimated by the interaction of odds ratio (IOR) in logistic regression models. If the 95% CI of IOR did not contain 1, the significant multiplicative interaction was considered to exist [39]. In order to evaluate additive interaction, the relative excess risk due to interaction (RERI) and the attributable proportion due to interaction (AP) were calculated [40]. In the absence of additive interaction, 95% CIs of RERI and AP were equal to 0 [39,41,42].

Furthermore, in order to explore whether breastfeeding mediated the association between maternal prenatal education attendance and autistic-like behaviors in preschoolers, a series of hierarchical logistic regressions were performed after adjusting for the potential confounders. According to Baron and Kenny [43], mediation is demonstrated when the following conditions are met: (1) the independent variable (X, maternal attendances to prenatal education) is significantly related with the dependent variable (Y, autistic-like behaviors of preschoolers; the partial coefficient is denoted by c); (2) the independent variable is significantly related to the mediator variable (M, breastfeeding; the partial coefficient is denoted by a ); and (3) the mediator variable (breastfeeding) is significantly associated with the dependent variable (autistic-like behaviors of preschoolers) when the independent variable (maternal attendances to prenatal education) is controlled for (the partial coefficient is denoted by b). The indirect effect of X on Y through M (namely OR^IE^) was estimated as exp(ab), and the direct effect of X on Y (namely OR^DE^) was estimated as exp(c’) (The c’ is the effect of X on Y controlling for M). Based on the research of Vanderweele [44], we used the following formula to calculate the proportion of mediation:

$$\frac{{\mathrm{OR}}^{\mathrm{DE}}\times \left({\mathrm{OR}}^{\mathrm{IE}}-1\right)}{\left({\mathrm{OR}}^{\mathrm{DE}}\times {\mathrm{OR}}^{\mathrm{IE}}-1\right)}$$

All of the *p*-values were two-sided. Type I errors were set at 0.05. The statistical analysis was conducted with SPSS (version 23.0; SPSS Inc., Chicago, IL, USA).

## 3. Results

### 3.1. Description of Participants

The descriptive statistics of the participants are presented in Table 1. Of the 67,578 successfully investigated children, 54.3% were male and the average age was 4.61 years (SD = 0.88) at the time of assessment, 90.2% were breastfed, 43.0% of the mothers never participated in prenatal education during pregnancy, 9.7% for once, 18.3% for twice, 9.8% for three times and 9.2% for four times and over. Of the children, 2074 (3.1%) were categorized with autistic-like behaviors.

### 3.2. Association between Maternal Participation in Prenatal Education and Demographic Characteristics

As shown in Table 2, the results of Chi-square tests and ANOVA revealed that those who attended maternal prenatal education differed to those who did not attend maternal prenatal education on a range of demographic characteristics. Pregnant women from families with high monthly incomes participated in prenatal education more often than those from families with low monthly incomes. Women who attended more often prenatal education tended to have female infants, older maternal/paternal age in years at child’s birth, higher maternal education level and higher paternal education level. However, there was no statistically significant relationship between maternal prenatal education attendance and marital status (*p* > 0.05).

### 3.3. Association between Autistic-Like Behaviors of Preschoolers and Demographic Characteristics

Table 3 shows that being a male child, low maternal education level, low paternal education level, low monthly family income and non-married status were significantly associated with a higher probability of the presence of autistic-like behaviors in preschool children (*p* < 0.001).

### 3.4. Association between Maternal Participation in Prenatal Education and Breastfeeding

After adjusting for the child’s age and gender, parental education level, monthly family income and parental marital status and age at child’s birth, the results of the logistic regression analysis showed that maternal participation in prenatal education was significantly associated with an increase in breastfeeding, see Table 4.

### 3.5. Association of Maternal Participation in Prenatal Education and Breastfeeding with Autistic-Like Behaviors of Preschoolers

Table 5 displays results of the binary logistic regression analyses examining the associations between the independent variables and prevalence of autistic-like behaviors. After controlling for the aforementioned covariates, the analysis indicated that maternal participation in prenatal education and breastfeeding (OR = 0.773, 95% CI = 0.676–0.884) were significantly associated with the less autistic-like behaviors in preschoolers. Compared with those who had never participation in prenatal education, the odds ratio of exhibiting autistic-like behaviors was 0.798 (95% CI = 0.707–0.901) for once, 0.738 (95% CI = 0.648–0.841) for twice, 0.732 (95% CI = 0.615–0.871) for three times and 0.738 (95% CI = 0.681–0.883) for four times and over. 

Moreover, the crossover analysis indicated that children with both maternal prenatal education and breastfeeding had a lower risk of presence of autistic-like behaviors (OR = 0.569, 95% CI = 0.478–0.677), and there was no multiplicative and additive interaction of two variables on autistic-like behaviors. See Table 6.

### 3.6. Mediating Effect of Breastfeeding on Maternal Participation in Prenatal Education and Autistic-Like Behaviors of Preschoolers

**Figure 1:**
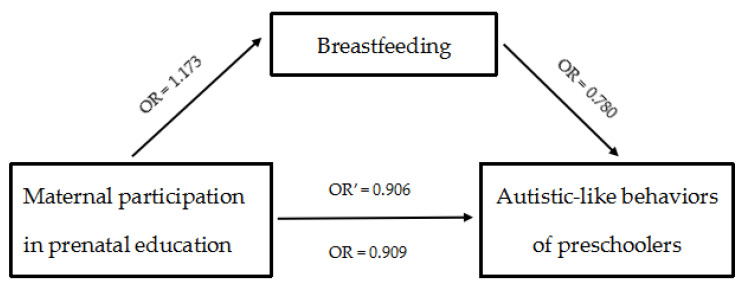
Steps of mediation analysis.

After controlling for the aforementioned confounders, the results from the binary logistic regression analysis (Table 7 and Figure 1) showed that maternal participation in prenatal education was significantly associated with decreased the probability of the presence of autistic-like behaviors (OR = 0.909, 95% CI = 0.876–0.942; Model 1 in Table 7). After breastfeeding was added into Model 1, the association between maternal participation in prenatal education and autistic-like behaviors became weaker, but was still significant (OR = 0.906, 95% CI = 0.873–0.939; Model 2 in Table 7). The mediation analysis indicated that breastfeeding mediated the association between maternal participation in prenatal education and autistic-like behaviors in preschoolers, with its effect being approximately 14.3%.

## 4. Discussion

To investigate the effect of maternal prenatal education attendance on autistic-like behaviors in preschoolers and its potential pathway, we conducted a cross-sectional study with 67,578 preschool children and their mothers from Longhua New District of Shenzhen, China. After adjusting for the child’s gender, parental educational level, family income and parental marital status, we found that attending prenatal education during pregnancy and breastfeeding was significantly associated with a reduced probability of preschoolers’ autistic-like behaviors. Moreover, crossover analysis indicated that compared with children without both maternal prenatal education and breastfeeding, those with both maternal prenatal education and breastfeeding had a lower risk of the presence of autistic-like behavior, followed by ones with maternal prenatal education only and one with breastfeeding only. And, we found no significantly multiplicative and additive interaction of prenatal education and breastfeeding on autistic-like behaviors in preschoolers. Furthermore, mediation analysis indicated that breastfeeding mediated in part the association between the maternal participation in prenatal education and autistic-like behaviors in preschoolers, with a mediating effect being around 14.3%.

### 4.1. Association between Maternal Participation in Prenatal Education and Breastfeeding

It has been well documented that prenatal education can substantially provide health promotion content for pregnant women and lead to them gaining positive attitudes and healthier behaviors during pregnancy [24,25,26,45,46]. For example, two prior studies showed that prenatal education could effectively avoid maternal exposure to detrimental environmental factors and inappropriate behaviors [20,23]. Prenatal education has positive effect on maternal folic acid and vitamin D intake [47], parental-infant interaction and maternal psychological stress [48], etc. Further, several investigations reported that attendance at group prenatal classes could increase the rate of exclusive breastfeeding [49,50,51]. Similarly, studies by Sehhatie et al. [52] and McFadden et al. [53] revealed that prenatal breastfeeding counseling increased the rates of exclusive breastfeeding. Yan presents evidence that the lower frequency of prenatal education visits, the higher risk of no breastfeeding [51]. In line with previous studies, our findings also support a positive association between maternal participation in prenatal education and breastfeeding. Collectively these findings indicate that maternal participation in prenatal education may increase the rate of breastfeeding.

### 4.2. Association between Breastfeeding and Autistic-Like Behaviors

Regarding effect of breastfeeding on ASD, a multicenter study in Spain found that breastfeeding played a protective role against autistic traits [54]. A case-control study involving 861 children with ASD and 123 controls showed that children who were not breastfed for more than six months were 2.48 times more likely to have autistic spectrum disorder compared to those who were breastfed for more than six months [55]. Moreover, another case-control study found that the optimal breast-feeding practices, including the early initiation of breastfeeding, increased periods of exclusive breastfeeding and continued breastfeeding as well, could decrease the risk of ASD [56]. In line with these previous findings, we also found a significant inverse relationship between breastfeeding and the presence of autistic-like behaviors in preschool children. Taken together, these findings support the premise that breastfeeding can reduce the risk of ASD or autistic-like behaviors in children.

### 4.3. Association between Maternal Participation in Prenatal Education and Autistic-Like Behaviors of Preschoolers and Potential Pathways

There are few studies examining the long-term effects of maternal attendance in prenatal education upon children’s health. Our study adds to the literature by showing that maternal attendance in prenatal education was significantly and negatively associated with the presence of autistic-like behaviors in preschoolers. Furthermore, mediation analysis indicated that breastfeeding mediated this association, with an effect of around 14.3%. How do we explain breastfeeding mediating the association between maternal attending prenatal education and autistic-like behaviors in preschoolers? Breastfeeding might act as a protective factor through two mechanisms [57]. First, from a nutritional standpoint, human breast milk contains a range of nutrients, such as carbohydrates, protein, fat, vitamins, minerals, hormones and immune cells, which can support the emotional and cognitive development of infants and protects them from contagious and chronic diseases [58]. These nutrients including iron [59], insulin-like growth factor (IGF) [60,61], long-chain polyunsaturated fatty such as acids docosahexaenoic (DHA) and arachidonic (ARA) acids improve infants’ cognitive development and may protect against autistic traits [62]. Compared with bovine milk or infant formula, human milk could elevate more serum IGF in neonatal [63,64]. And the IGF could improve myelination, which can promote more effective neural impulse passage [60]. The physiological effects of IGF-1 are included stimulation of neuro cell proliferation, promotion of tissue growth and development, effects on lipid and carbohydrate metabolism, and anti-inflammatory and anti-oxidant effects as well [65]. In addition, the dietary DHA enrichment of human milk could have an effect on improving neurodevelopment only when mothers start supplementation during pregnancy [66]. Second, prenatal education could increase maternal-fetal attachment and self-efficacy related to childbirth [67,68]. As a uniquely close and sensual experience, breastfeeding creates a particular connection between mother and neonatal, which promotes mother–infant attachment [69,70]. It is known that maternal stress during pregnancy may affect motor and mental development in infants, and the effect may last to later child development [71]. Also, postpartum maternal stress is negatively associated with child poor motor, language and cognitive development [72]. The production of prolactin and oxytocin during breastfeeding not only can increase maternal-fetal bonding and skin-to-skin contact, but also confers maternal relaxation benefits [73]. Liu et al. have found that breastfeeding and active maternal-fetal bonding are in relation to lower risks of internalizing behavior problems in age six mainly by promoting neurodevelopment in early childhood [74]. Nevertheless, the exact mechanism for the associations of autistic-like behaviors, breastfeeding and prenatal education needs to be further explored.

### 4.4. Strength and Limitation

To the best of our knowledge, our study is the first to show that maternal participation in prenatal education protects against the development of autistic-like behaviors in preschoolers. However, certain limitations of this study need to be noted. First, although the study sample size is very large, the study participants were all from Longhua New District of Shenzhen, China. This is a city with high levels of population mobility, which may limit the generalizability to other Chinese cities. Second, it is possible that the measures of attendance at prenatal education and engagement in breastfeeding may be influenced by recall bias. However, a couple of studies showed that mothers’ reports were important to improve the accuracy of retrospective data and may appear more reliable than medical records sometimes [75,76]. Third, while our study supports the protective effects of prenatal education against autistic-like behaviors, we did not use cut-offs to indicate the presence of ASD and so the result should not be interpreted as an indication that prenatal education prevents the development of ASD. Further examination of such processes in both clinical and sub-clinical ASD has the potential to further our understanding of the broader autism phenotype. Fourth, breastfeeding only partially mediated the relations between maternal participation in prenatal education and children’s autistic-like behaviors, and the effect is relatively small. This indicates that other possible pathways may exist and need to be further explored. Fifth, we did not ask the participants to respond to breastfeeding via pumped milk whether or not, which had different impacts than feeding baby at the breast [77]. This might disturb the true association between breastfeeding and autistic-like behaviors in our results. In addition, the exact mechanism for the associations of autistic-like behaviors in preschoolers, breastfeeding and prenatal education needs to be further explored. Finally, significant paths identified in mediation analysis do not establish causal effects in this cross-sectional study. Thus, further investigation in a longitudinal study is needed to confirm our findings.

## 5. Conclusions

In summary, our findings suggest that maternal participation in prenatal education is associated with a decreased risk of autistic-like behaviors in preschoolers, in part due to increasing breastfeeding. The findings reinforce the importance of promoting prenatal education, not only for the immediate beneficial effects on pregnant women and their infants, but also long-term benefit effects on their offspring’s health.

## Figures and Tables

**Table 1:** Sample characteristics (*n* = 67,578).

Characteristics	Total
Child age in years, mean (SD)	4.61 (0.88)
Gender, *n* (%)	
Male	36,669 (54.3)
Female	30,909 (45.7)
Maternal age in years at child’s birth, mean (SD)	26.99 (4.18)
Maternal education level, *n* (%)	
High school or lower	29,371 (43.5)
Technical secondary school or college	24,479 (36.2)
University	12,425 (18.4)
Postgraduate or higher	1303 (1.9)
Paternal age at child’s birth, mean (SD)	29.56 (4.76)
Paternal education level, *n* (%)	
High school or lower	26,486 (39.2)
Technical secondary school or college	21,810 (32.3)
University	16,951 (25.1)
Postgraduate or higher	2331 (3.4)
Monthly family income, *n* (%)	
< 5000 RMB	10,272 (15.2)
5000–15,000 RMB	31,587 (46.7)
15,000–25,000 RMB	14,459 (21.4)
≥ 25,000 RMB	11,260 (16.7)
Parental marital status, *n* (%)	
Married	65,278 (96.6)
Other ^a^	2300 (3.4)
Maternal participation in prenatal education, *n* (%)	
Never	29,067 (43.0)
Once	13,331 (19.7)
Twice	12,361 (18.3)
Three times	6591 (9.8)
≥Four times	6228 (9.2)
Breastfeeding, *n* (%)	
No	6632 (9.8)
Yes	60,946 (90.2)
Autistic-like behaviors of preschool children, *n* (%)	
No	65,504 (96.9)
Yes	2074 (3.1)

^a^ Including remarriage, divorce, widowed and unmarried.

**Table 2:** Maternal attendances to prenatal education in different socio-demographic characteristics ^#^.

Characteristics	Total (*n* = 67,578)	Participation in Prenatal Education					F/χ^2^	*p* Value
		Never (*n* = 29,067)	Once (*n* = 13,331)	Twice (*n* = 12,361)	Three Times (*n* = 6591)	≥Four Times (*n* = 6228)		
Child age in years, mean (SD)	67,578	4.60 (0.88)	4.61 (0.88)	4.62 (0.87)	4.59 (0.87)	4.61 (0.88)	1.306	0.265
Gender, *n* (% )							20.63	<0.001
Male	36,669	15,958 (43.5)	7207 (19.7)	6732 (18.4)	3420 (9.3)	3352 (9.1)		
Female	30,909	13,109 (42.4)	6124 (19.8)	5629 (18.2)	3171 (10.3)	2876 (9.3)		
Maternal age in years at child’s birth, mean (SD)	67,578	26.43 (4.20)	27.09 (4.02)	27.41 (4.11)	27.71 (4.14)	27.76 (4.15)	185.3	<0.001
Maternal education level, *n* (% )							1448.3	< 0.001
High school or lower	29,371	14,613 (49.8)	5512 (18.8)	4822 (16.4)	2352 (8.0)	2072 (7.1)		
Technical secondary school or college	24,479	9957 (40.7)	5003 (20.4)	4691 (19.2)	2497 (10.2)	2331 (9.5)		
University	12,425	4110 (33.1)	2576 (20.7)	2554 (20.6)	1548 (12.5)	1548 (12.5)		
Postgraduate or higher	1303	387 (29.7)	240 (18.4)	294 (22.6)	194 (14.9)	194 (14.9)		
Paternal age in years at child’s birth, mean (SD)	67,578	29.03 (4.80)	29.67 (4.58)	29.96 (4.72)	30.18 (4.70)	30.34 (4.76)	270.1	< 0.001
Paternal education level, *n* (%)							1537.1	< 0.001
High school or lower	26,486	13,455 (50.8)	4889 (18.5)	4226 (16.0)	2096 (7.9)	1820 (6.9)		
Technical secondary school or college	21,810	9053 (41.5)	4432 (20.3)	4118 (18.9)	2199 (10.1)	2008 (9.2)		
University	16,951	5851 (34.5)	3571 (21.1)	3491 (20.6)	1978 (11.7)	2060 (12.2)		
Postgraduate or higher	2331	708 (30.4)	439 (18.8)	526 (22.6)	318 (13.6)	340 (14.6)		
Monthly family income, *n* (%)							1200.7	< 0.001
<5000 RMB	10,272	5415 (52.7)	1743 (17.0)	1558 (15.2)	789 (7.7)	767 (7.5)		
5000–15,000 RMB	31,587	14,443 (45.7)	6230 (19.7)	5559 (17.6)	2809 (8.9)	2546 (8.1)		
15,000–25,000 RMB	14,459	5341 (36.9)	3066 (21.2)	2921 (20.2)	1662 (11.5)	1469 (10.2)		
≥25,000 RMB	11,260	3868 (34.4)	2292 (20.4)	2323 (20.6)	1331 (11.8)	1446 (12.8)		
Parental marital status, *n* (%)							8.782	0.067
Married	65,278	28,075 (43.0)	12,897 (19.8)	11,950 (18.3)	6378 (9.8)	5978 (9.2)		
Other ^a^	2300	992 (43.1)	434 (18.9)	411 (17.9)	213 (9.3)	250 (10.9)		

^#^*χ*^2^ tests were used for categorical variables, ANOVA were used for continuous variables. ^a^ Including remarriage, divorce, widowed and unmarried.

**Table 3:** Autistic-like behaviors of preschool children in different socio-demographic characteristics ^#^.

Characteristics	Total (*n* = 67,578)	Autistic-Like Behaviors		t/χ^2^	*p* Value
		No (*n* = 65,504)	Yes (*n* = 2074)		
Child age in years, mean (SD)	67,578	4.62 (0.87)	4.34 (0.89)	14.11	< 0.001
Gender, *n* (% )				64.658	< 0.001
Male	36,669	35,364 (96.4)	1305 (3.6)		
Female	30,909	30,140 (97.5)	769 (2.5)		
Maternal age in years at child’s birth, mean (SD)	67,578	27.02(4.16)	25.09(4.43)	12.08	< 0.001
Maternal education level, *n* (%)				179.307	< 0.001
High school or lower	29,371	28,195 (96.0)	1176 (4.0)		
Technical secondary school or college	24,479	23,823 (97.3)	656 (2.7)		
University	12,425	12,198 (98.2)	227 (1.8)		
Postgraduate or higher	1303	1288 (98.8)	15 (1.2)		
Paternal age at child’s birth, mean (SD)	67,578	29.59 (4.74)	28.58 (5.07)	9.54	< 0.001
Paternal education level, *n* (%)				188.564	< 0.001
High school or lower	26,486	25,390 (95.9)	1096 (4.1)		
Technical secondary school or college	21,810	21,212 (97.3)	598 (2.7)		
University	16,951	16,610 (98.0)	341 (2.0)		
Postgraduate or higher	2331	2292 (98.3)	39 (1.7)		
Monthly family income, *n* (%)				189.948	< 0.001
<5000 RMB	10,272	9806 (95.5)	466 (4.5)		
5000–15,000 RMB	31,597	30,508 (96.6)	1089 (3.4)		
15,000–25,000 RMB	14,459	14,137 (97.8)	322 (2.2)		
≥25,000 RMB	11,260	11,063 (98.3)	197 (1.7)		
Parental marital status, *n* (%)				17.917	< 0.001
Married	65,278	63,309 (97.0)	1969 (3.0)		
Other ^a^	2300	2195 (95.4)	105 (4.6)		

^#^*χ*^2^ tests were used for categorical variables, t tests were used for continuous variables. ^a^ Including remarriage, divorce, widowed and unmarried.

**Table 4:** Association between maternal participation in prenatal education and breastfeeding ^#^.

Maternal Participation in Prenatal Education	Breastfeeding		OR (95% CI)	*p* Value
	No (*n* = 6632)	Yes (*n* = 60,946)		
Never (*n* = 29,067)	3337 (11.5)	25,730 (88.5)	1.00	
Once (*n* = 13,331)	1325 (9.9)	12,006 (90.1)	1.191 (1.113–1.274)	< 0.001
Twice (*n* = 12,361)	1042 (8.4)	11,319 (91.6)	1.435 (1.333–1.545)	< 0.001
Three times (*n* = 65,911)	521 (7.9)	6070 (92.1)	1.539 (1.396–1.697)	< 0.001
≥Four times (*n* = 6228)	407 (6.5)	5821 (93.5)	1.899 (1.705–2.115)	< 0.001

^#^ With adjustment for child’s age, child’s gender, parental education level, parents’ age at child’s birth, monthly family income and parental marital status.

**Table 5:** Association of maternal participation in pregnancy school variables and breastfeeding with autistic-like behaviors of preschool children ^#^.

Variables	Total (*n* = 67,578)	Autistic-Like Behaviors		OR (95% CI)	*p* Value
		No (*n* = 65,504)	Yes (*n* = 2074)		
Maternal participation in pregnancy school					
Never	29,067 (43.0)	27,954 (96.2)	1113 (3.8)	1.00	
Once	13,331 (19.7)	12,966 (97.3)	365 (2.7)	0.798 (0.707–0.901)	<0.001
Twice	12,361 (18.3)	12,060 (97.6)	301 (2.4)	0.738 (0.648–0.841)	<0.001
Three times	6591 (9.8)	6439 (97.7)	152 (2.3)	0.732 (0.615–0.871)	<0.001
≥Four times	6228 (9.2)	6085 (97.7)	143 (2.3)	0.738 (0.681–0.883)	0.001
Breastfeeding					
No	6632 (9.8)	6375 (96.1)	257 (3.9)	1.00	
Yes	60,946 (90.2)	59,129 (97.0)	1817 (3.0)	0.773 (0.676–0.884)	<0.001

^#^ With adjustment for child’s age, child’s gender, parental education level, parents’ age at child’s birth, monthly family income and parental marital status.

**Table 6:** The combination effect of maternal participation in prenatal education and breastfeeding on autistic-like behaviors in pre-schoolers ^#^.

Variables		Autistic-Like Behaviors OR (95% CI)	IOR (95% CI)	RERI (95% CI)	AP (95% CI)
Prenatal education	Breastfeeding		1.175 (0.891, 1.594)	0.176 (−0.027, 0.378)	0.309 (−0.072, 0.690)
No	No	1.000			
Yes	No	0.664 (0.512, 0.861) *			
No	Yes	0.729 (0.614, 0.866) **			
Yes	Yes	0.569 (0.478, 0.677) **			

^#^ With adjustment for child’s age, child’s gender, parental education level, parents’ age at child’s birth, monthly family income and parental marital status. * *p* = 0.002; ** *p* < 0.001.

**Table 7:** Mediating effect of breastfeeding on association between maternal participation in prenatal education and autistic-like behaviors of preschoolers ^#^.

Characteristics	Autistic-Like Behaviors (Dependent Variable, Y, OR, 95% CI)		
	Model 1 X → Y	Model 2 X + M → Y	Proportion of Mediation
Maternal participation in pre-natal education (Independent Variable, X)	0.909 (0.876, 0.942) *	0.906 (0.873, 0.939) *	14.3%
Breastfeeding (Mediator, M)		0.780 (0.681, 0.892) *	

^#^ With adjustment for child’s age, child’s gender, parental education level, parents’ age at child’s birth, monthly family income and parental marital status. * *p* < 0.001.

## Data Availability

The data presented in this study are available on request from the corresponding author. The data are not publicly available due to privacy concerns.
